# Organization-level principles and practices to support spiritual care at the end of life: a qualitative study

**DOI:** 10.1186/s12904-017-0197-9

**Published:** 2017-04-11

**Authors:** Paul Holyoke, Barry Stephenson

**Affiliations:** 1 Saint Elizabeth Research Centre, Saint Elizabeth Health Care, 90 Allstate Parkway, Suite 300, Markham, ON Canada L3R 6H3; 2 Department of Religious Studies, Memorial University of Newfoundland, St. John’s, NF Canada A1A 5S7

**Keywords:** Spiritual care, Palliative care, Interdisciplinary team, Best practices, Secular health care, Volunteers, End of life care

## Abstract

**Background:**

Though most models of palliative care specifically include spiritual care as an essential element, secular health care organizations struggle with supporting spiritual care for people who are dying and their families. Organizations often leave responsibility for such care with individual care providers, some of whom are comfortable with this role and well supported, others who are not. This study looked to hospice programs founded and operated on specific spiritual foundations to identify, if possible, organizational-level practices that support high-quality spiritual care that then might be applied in secular healthcare organizations.

**Methods:**

Forty-six digitally-recorded interviews were conducted with bereaved family members, care providers and administrators associated with four hospice organizations in North America, representing Buddhist, Catholic, Jewish, and Salvation Army faith traditions. The interviews were analyzed iteratively using the constant comparison method within a grounded theory approach.

**Results:**

Nine Principles for organizational support for spiritual care emerged from the interviews. Three Principles identify where and how spiritual care fits with the other aspects of palliative care; three Principles guide the organizational approach to spiritual care, including considerations of assessment and of sacred places; and three Principles support the spiritual practice of care providers within the organizations. Organizational practices that illustrate each of the principles were provided by interviewees.

**Conclusions:**

These Principles, and the practices underlying them, could increase the quality of spiritual care offered by secular health care organizations at the end of life.

**Electronic supplementary material:**

The online version of this article (doi:10.1186/s12904-017-0197-9) contains supplementary material, which is available to authorized users.

## Background

In caring for people with terminal illness, needs, concerns, and questions of a religious, spiritual, or existential nature are usually present [1]. Spiritual care, broadly conceived, is necessary for the person who is dying, but also for family members and care providers.

The origins of contemporary hospice care in the work of Dame Cicely Saunders in the post WWII decades was rooted in the recognition of the complex, multidimensional nature of end of life. Hospice care, Saunders understood, includes much more than the management of physical pain. Saunders speaks of ‘total pain,’ consisting of physical, psychological, emotional, and spiritual dimensions [2]. The ‘holistic’ model inaugurated by Saunders explicitly or implicitly shapes much of the current discussions and conceptions of hospice palliative care —and yet there remains a significant gap between theory and practice, between acknowledged need and delivered care.

The medical model has been the dominant paradigm in health care for over a century. Historically, conceptual and institutional developments in modern medicine have been premised on the exclusion of religious (or spiritual) attitudes, ideas, and practices. During the same period, health care has become almost exclusively the provenance of the modern western state, which, like modern medicine, defined itself in part by excluding religion and spirituality from its business [3].

The fact that both the medical model and the organization and delivery of health care have distanced themselves from religion has led to a situation where, at the practical level, it is recognized that spiritual care is required at end of life, but at the organizational, managerial, and cultural level, there are few supports for actually delivering such care. Recent studies of nursing leadership, for example, revealed that the recognition of the spiritual dimension of care in nursing exists alongside a cautious reluctance within managerial culture to integrate spirituality into nursing practice [2, 4, 5]. At the level of patient and family experience, a recent assessment of palliative care in Nova Scotia demonstrated that ‘[c]ommunication in various forms and family emotional and spiritual support were consistently viewed [by next of kin] as lacking in all locations and identified as targeted areas for impacting quality care at end of life.’ [6] These findings are representative of an issue that has emerged in hospice palliative care planning, delivery, and research: there is need to improve the spiritual dimension of care, but historical, cultural, educational, political, and other contexts surrounding and informing the delivery of care inhibit integrating the spiritual into end of life care [7–13].

The disjunction between acknowledged need for spiritual care and on-the-ground organization and practice generated the central question driving this study:

How can hospice palliative care – which recognizes the importance of spiritual care at the end of life and beyond, yet which is chiefly framed and informed by secular ideas, contexts, and practices – better meet the religious, spiritual, and existential questions and needs often present at end of life?

Since, practically speaking, most hospice palliative care in the foreseeable future will continue to be organized and practiced chiefly within or connected to health care organizations not founded and operated on specific spiritual foundations in Canada and other countries, there is a need to find ways to incorporate religion and spirituality into the organization and delivery of care offered in these secular settings. This will help to meet the needs of people who are dying, their family members, and, often, their care providers^1^ live up to the intentions informing statements of best practice in hospice palliative care.

One possible way to enhance the provision of spiritual care would be to focus on the contributions of individual care providers [14, 15] or specialist spiritual care providers [16]. However, experience shows that without organization-wide support for individual care providers’ efforts, depending solely on the motivation and abilities of individual providers leads to mixed results [17].

Another way to reflect on reorienting hospice palliative care in chiefly secular settings is to explore whether valuable lessons may be learned from the organization and delivery of care practiced at hospice organizations that have an explicit religious or spiritual orientation, committed as they are to the spiritual dimensions of end of life care. This is the approach taken in this study, with a focus on organizational level practices and approaches that could support and complement the efforts of individual care providers. An assumption guiding this research project was that there is a set of useful, generalizable ideas and practices in place in these organizations contextualized with specific religious traditions, and these ideas and practices might be transferred or translated to more or less secular hospice palliative care settings.

## Methods

This study employed qualitative methods rooted in the grounded theory tradition. Grounded theory is a qualitative research method that allows a situation or phenomenon to be examined in order to understand how key players manage their roles and then, through inductive reasoning, develops a theory or framework in order to convey an understanding about the situation or phenomenon. In this study, data collection and analysis follow the grounded theory constant comparative approach [18, 19], viewing data collection and analysis as a single concurrent process in which the method is fluid and evolves as the understanding unfolds from the data.

Over the course of several months in 2014, the authors visited four hospice organizations, each informed by an explicit religious context: the Catholic Hospice in Miami, Florida; the Zen Hospice Project in San Francisco, California; the Salvation Army Agapé Hospice in Calgary, Alberta; and, the Jewish Hospice Program of Jewish Family & Child in Toronto, Ontario. Though framed and informed by religious traditions, each of these hospice programs is inclusive, serving a diverse range of persons. One need not be Catholic, Buddhist, Christian or Jewish to access their services.

The Catholic Hospice operates a home-based program and a residential hospice facility with 20 beds. The Zen Hospice Project and the Salvation Army Agapé are residential hospices with 6 and 20 beds respectively, and the Zen Hospice Project collaborates on providing volunteers for the hospice program at Laguna Honda Hospital in San Francisco. The Toronto Jewish Hospice Program is a home-based program, with a chaplain, social workers, and counsellors from Jewish Family & Child Services of Greater Toronto and volunteers coordinated by Circle of Care, a multi-service organization.

The study was conducted through semi-structured interviews with staff, volunteers, and bereaved family members at these hospice palliative care programs. The profile of the interviewees is provided in Table 1. The questions that guided the interviews focused on the interviewees’ perceptions of the supports they receive or expect or request from their hospice organization in the provision of spiritual care (the question sets are in Additional file 1). The interviews were documented using digital video and, when the interviewees specifically requested it (three did), digital audio replaced the use of video.

**Table 1:** Profile of interviewees

	Number
Gender	
Female	36
Male	10
Role	
Staff	30
Chaplain/Spiritual care provider	4
Health care provider	14
Adminstrator	12
Volunteer	16
Family member	9
Location	
Catholic Hospice, Miami	13
Zen Hospice Project, San Francisco	11
Salvation Army Agapé Hospice, Calgary	15
Jewish Hospice Program, Toronto	7

The researchers conducted the interviews together at the first two hospices, the Catholic Hospice and the Zen Hospice Project, and one researcher (PH) conducted the interviews at the others. After conducting each interview (and for the later hospice interviews, after BS reviewed the interview recordings), the researchers separately recorded field notes and reflected, first separately and then collaboratively. From the field notes and the reflections, lists and descriptions of organization-level best practices were first developed by the researchers separately and then evolved into a set of general themes (Principles) and practices through discussion. After all the interviews were completed, both researchers reviewed all the digital recordings and revised the Principles, and specific illustrative quotations from several interviews were assembled in a series of short videos, one video for each Principle. The written version of Principles and practices led to other revisions, and the short videos were re-edited to reflect the newest analysis. The Principles were then re-ordered into three categories or meta-themes, to help with conceptual organization and translation of the results. Some of the preliminary results were presented in conference settings, which provided other occasions for revisions.

Both theoretically and methodologically a key issue informing research on end of life care is how best to define and conceptualize ‘spiritual care.’ Sinclair and colleagues [20] found that the definition is a dominant issue in the research literature, and that there are three main approaches to the definition question: (1) some researchers assume that no definition is possible because the meaning of ‘spiritual care’ and ‘spirituality’ are beyond the capabilities of words, or simply too nebulous and broad; (2) others establish as broad a definition as possible, including as many general concepts as possible to include all possible interpretations; and, (3) others adopt a narrow definition that assists in locating aspects of spiritual care or that conforms to their research interests or personal belief system.

In this study, the method aligned with the second approach: the interview questions were posed in a neutral way that enabled interviewees to respond with their own definitions of ‘spiritual’ and ‘spiritual care’ in mind. In many of the interviews at the hospice programs, care was variously described as ‘spiritual,’ ‘religious,’ ‘existential,’ ‘psychosocial,’ ‘human,’ and ‘meaningful.’ This approach parallels the second Principle (see below), which emphasizes the agency of the dying person and his or her family – rather than the care providers – to guide and direct the kinds of spiritual care that is needed.

## Results and discussion

On the basis of the interviews and the subsequent analysis, three ‘foundational’ Principles of quality end of life spiritual care, and six ‘enabling’ Principles were identified (Table 2). The first three Principles are necessary for the other Principles to be put into practice. In the following, each Principle is formulated in a clear statement, integrating the words and ideas gathered from interviewees, and then the features of each Principle are described. Suggestions of how organizations may realize these principles in practice are also provided.

**Table 2:** The Nine Principles

Foundational principles that influence the organization of spiritual care 1. Quality spiritual care incorporates the spiritual into every other aspect of hospice palliative care such that the spiritual is not merely a part or element of care, but rather a descriptor of the kind, nature and quality of all care. 2. More profoundly than in any other area of care, quality spiritual care is guided and directed by the dying person and the family. 3. Hospice palliative care is fundamentally a vocation, and the work is inherently spiritual.	
Principles that enable a high‐quality approach to spiritual care by care providers 4. Quality spiritual care requires care providers to allow spiritual questions and issues to emerge. 5. Quality spiritual care entails the act of ‘witnessing.’ 6. Quality spiritual care considers place as sacred.	
Principles that enable the spiritual care practices of care providers 7. Quality spiritual care includes rituals and times dedicated to marking transitions and processing experiences. 8. Quality spiritual care involves creating and sustaining relationships beyond those typical between co‐workers. 9. Quality spiritual care emphasizes the role of volunteers, whose presence and work reinforces and ensures that hospice palliative care is grounded as vocational and spiritual.	

### Principle 1

Quality spiritual care incorporates the spiritual into every other aspect of hospice palliative care such that the spiritual is not merely a part or element of care, but rather a descriptor of the kind, nature, and quality of all care.

The hospices visited are rooted in specific religious traditions; in conceptualizing the spiritual dimensions of end of life, these hospices do not view spirituality and religious concerns as an element or component of overall palliative care; rather, spiritual care is *embedded* throughout the giving of care. The word ‘spiritual’ in the term ‘spiritual care’ does not refer to one among several specialized treatment or care domains (for example, social, psychological, spiritual); rather, the spiritual emerges as the tone, mood, or atmosphere of the care being given.

Current models of hospice palliative care recognize that a range of issues arise in the course of life-limiting illness and processes of bereavement. Hospice palliative care is often conceptualized in terms of a number of distinct though inter-related ‘domains’ or ‘dimensions.’ One of the domains or dimensions of hospice palliative care is the ‘spiritual,’ and models of best practices generally acknowledge the vital role of spirituality in hospice palliative care [21–23]. The list of domains of issues identified by the Canadian Hospice Palliative Care Association [23] includes Physical, Disease Management, Psychological, Loss/Grief, Social, End of Life Care/Death Management, Practical, and Spiritual; Patient and Family Characteristics are depicted as being at the centre of the other domains. The Canadian Hospice Palliative Care Association’s *A Model to Guide Hospice Palliative Care*, states that ‘[i]f one or more issues are missed, they can compound one another, leading to distress.’ [23] Similarly, in the *Spiritual Care Nation-wide Guideline* from the Netherlands, a model is presented that places “spiritual problems” at the centre of a circle, with interactions with the psychosocial and physical problem domains surround the spiritual domain.

Furthermore, it has been shown that the spiritual dimension interacts with other domains. Attention to the spiritual has been shown to reduce psychological suffering [24]; those with higher spiritual well-being suffer fewer physical symptoms such as cancer-related fatigue [25]; and, having a religious tradition or spiritual practice correlates with the experience of a better quality of life [26].

However, the findings from the hospices in this study point to something beyond the interaction of distinct domains. Life closure events (closing relationships or saying goodbye) are not only in the ‘death management’ domain as in the Canadian Hospice Palliative Care Association’s model; tending to anticipatory grief is not only in the ‘loss, grief’ domain; assisting in personal hygiene is not only in the ‘practical’ domain. Each element of hospice palliative care has the potential to be interpreted, provided, and received in spiritual terms, and with a spiritual intent or spiritual impact.

One interviewee, a rabbi, expressed the view that spirituality and spiritual care need to be seen as another part of the ‘toolbox’ of care to offer to people who are dying, rather than a ‘treatment here and a treatment there.’ The metaphor used by the rabbi points to a crucial issue in articulating and implementing best practices. On one hand, the rabbi’s imagery of a care toolbox suggests that spiritual care is a particular thing, a particular tool, distinct from other tools, and one can reach in, take it out, and apply it to the situation. On the other hand, his refusal to think about spiritual care as ‘a treatment here and a treatment there’ points to a more diffuse sense of the spiritual in which spiritual care is not a piece or a part but rather a fundamental quality of what transpires in palliative care.

Thus, spiritual care is not something peripheral, distinct, or separate from other kinds of care; rather, spiritual care describes the nature of the care itself, not merely a specialized aspect of that care. The language of dimensionality is different from the language of modelling. A model has pieces, parts that fit together in a certain way, and models represent in miniature some larger structure or pattern. But models are also developed as a kind of blueprint for creating the real thing. Dimensionality points to that within which we live; a dimension is not a post in a field, but the field itself, not part of a model but the ‘space’ that model inhabits. Just as we exist in space and time, care at the end of life exists within the dimension of the spiritual. The evidence from the interviewees shows that models of care that include the spiritual as only one of its parts (even though they may refer to the part as a dimension) are necessary to best practices, but they are also insufficient.

The executive director of the Catholic Hospice said that in providing care to those who are dying, there needs to be a readiness to attend to spiritual needs when the needs arise – in place of, or in conjunction with, other care. She said,

whatever is needed … needs to be provided. We can call in assistance but between the time a challenge comes up – if someone asks you to pray at their bedside, you don’t say, “no, wait, let’s get the chaplain to do that.” We don’t do that. Everyone has the power to be able to sit down and have that prayer, or to read the Bible if they choose, or to read the Qur’an if they would like us to. Whatever that is, we’re there to be able to do that.

A nurse at the Zen Hospice Project illustrated the weaving together of different parts of care when she noted that administering pain medication is not merely a biomedical act, but a spiritual act of compassion, sympathy, and love:

Sure you have to do the medical, but it’s more than that. You have to give of your spirit.... I am not just going to give medications, it’s more than that. I have to put something behind that, in the way I administer it, and how I approach the bedside with it, and involve the family, and not be rushed, just be very slow… if I do that then I would say it is part of a spiritual practice.

Principle 1 therefore sees the spiritual as an emergent property of the system of care, not a distinct piece or part of care. Such a Principle has implications for the widely deployed team approach to palliative end of life care, the interprofessional care team, comprised of individuals each of whom contributes to the care with specialized knowledge and skills relevant to their respective domain, gained and certified by formal education and licensing or professional associations. The Canadian Hospice Palliative Care Association’s *A Model to Guide Hospice Palliative Care* [21] describes interprofessional care in this way:

Hospice palliative care is most effectively delivered by an interprofessional team of health care providers who are both knowledgeable and skilled in all aspects of care *within their discipline of practice*. (emphasis added)

This interprofessional approach recognizes and tends to respect and replicate multiple sectors of care, understanding that the patient and the family feel the cumulative effect.

The evidence from the hospice programs in this study, however, provides a different perspective. Not all care providers must be ‘expert’ in spiritual care, as measured against a lifetime of practice or study of religious and spiritual issues, or a specialization in a faith tradition. However, all must be prepared, when a spiritual issue arises or could or should arise, to respond to it. A staff member at the Catholic Hospice said that most care providers,

… have an advantage over the chaplain, the priest, the rabbi, the imam, because they are considered religious persons. They have their place but they tend to wait until the end when there’s nothing else to do. Nurses …. and myself are there the first time and we are welcomed with open arms.

A similar idea was expressed by an interviewee (a volunteer) at the Zen Hospice Project:

Every human being… if they can tap into it, has this human ability to be chaplain-like. People have been dying for as long as there have been people, and so there have been people sitting next to them for as long, you know, and I think we all have that ability. People think they don’t. People say, “You do such great work,” and I’m like, “It’s nothing different from what everyone else could do.” There’s no magic.

One of the interviewees at the Catholic Hospice described her experience as a family member:

You know what? I was very close to my mother’s nurse and she brought me more comfort than the priest that came, who I didn’t really know all that well. I knew the nurse and the aide that bathed my mother every day.

This last comment echoes the words of Lunn [27], who notes that

religious issues … are, like most issues of belief, very personal and as these questions are usually addressed to us directly as an individual regardless of profession they must be answered as such…. The shorter, less formulated, response of the nurse for example is more comforting and true for people than that of the “professional”.

Curlin and Hall [28] argue against a conception of spiritual care as a technique or skill acquired through technical training, possessed by certified professionals. They would rather hospice palliative care use the language of ‘wisdom’ and ‘moral friendship.’ In his research on the most valued components of care for the dying person and family, Chochinov [29] writes of the ‘ABCDs of Dignity Conserving Care.’ He refers to a set of attitudes and behaviors informed by compassion and dialogue, and holds that this expression and embodiment of dignity should be within the capabilities of all care providers.

An individual who approaches hospice palliative care vocationally (see Principle 3) likely possesses the capacities necessary to respond to spiritual needs. Those with specialized training may have a different kind of knowledge, which may be needed in specific situations; for example, a priest is needed to deliver last rites, or if a patient has been struggling for several weeks with existential searching and a chaplain or a psychologist may be needed [30]. The Netherlands’ *Spiritual Care Nation-wide Guideline* [30] says that doctors and nurses should, possibly with some additional training, be capable of most of the aspects of spiritual care – attention, counselling, and even crisis intervention, as long as the crisis does not last longer than a few days or a few weeks [30]. The approach by Marie Curie Cancer Care to spiritual and religious care competencies affirms this concept:

Although spiritual care is traditionally seen as the chaplain’s area of expertise these competencies assume that all staff and volunteers can and do provide spiritual care. ([31]:2)

Being available and having the time to build a necessary trust, all care providers can become the sources of spiritual comfort and the sounding boards for spiritual inquiries of the dying person and his or her family. This Principle, then, goes beyond integration, if integration means that different aspects of care are complementary and coordinated in a way that patients and their families find agreeable [11, 32–34]. Therefore, this Principle requires not only an adjustment to the depiction of the domains of palliative care, but also to move the concept of a care team composed of a group of health care providers with complementary but exclusive disciplines to one in which there is responsibility for spiritual care across the whole team.

The requirement to contribute to spiritual care may be a burden on a provider if he or she does not feel qualified or prepared to provide this kind of care; and sometimes, the person in need of spiritual care prefers a particular care provider [35]. In the latter case, there is clearly a need to find and bring forward the preferred provider. In the former case, the organization needs to address feelings of lack of preparedness, by providing an orientation to spiritual care that can be delivered by all members of the team. Many of the interviewees spoke of the need for ‘spiritual literacy,’ including a familiarity with common beliefs and practices of various faith traditions. The nature of the spiritual care that this Principle is addressing, however, is a broader issue. Principles 4, 5, and 6, provide means for equipping care providers to provide spiritual care, and with the confidence to do so. Properly equipping care providers also entails a system and processes to protect against, and deal with, situations when they feel overwhelmed by an expressed need, or when they discern that they are not able to meet a person’s spiritual needs, or when they themselves are in need of encouragement or spiritual care. Principles 7, 8 and 9 provide guidance on best organizational practices for these systems and processes.

### Principle 2

More profoundly than in any other area of care, quality spiritual care is guided and directed by the dying person and the family

It is clearly the best practice for the person who is dying to make decisions about the care that he or she is receiving or will receive as death approaches [23, 36, 37]. Of course, when it comes to illness and disease and their treatment, the knowledge and skills of experts are highly valued. But dying is, of course, no illness, but rather a part of life, and there are no experts in living—or, if there are, such expertise may well be found among the dying. The evidence from the interviewees in the hospices in this study strongly confirmed that the dying person takes the lead in spiritual discussions and the spiritual care work.

At the Zen Hospice Project, one interviewee, an administrator, emphasized that,

we have a radical acceptance or radical openness to meeting the resident right where they are… it’s really about letting the resident lead the experience. It doesn’t matter what happened last week. It doesn’t matter what we want. If we’re holding any agenda, like, drop it at the door. It’s really about letting the resident lead the experience and being open to whatever they need from us.

A nurse at the Catholic Hospice said that she hands over to the dying person the ability to name what care is needed:

If … you go in with God in your heart and in your hands, and you forget everything else, anything else, and you just concentrate on the patient and family, like ‘how can I do to help you?’, ‘what else can I do to help you?’

Another interviewee, a rabbi, said that, as a spiritual care provider, he is prepared to play different roles, depending on the dying person’s needs at any specific time:

I can come in as a rabbi if they need a religious authority, I can be a teacher if they need to know what Jewish traditions say. I can be a friend if, because of the terminal illness, the friends have been scared away. I will let the person drive the bus.

A volunteer interviewee at the Zen Hospice Project described building rapport in order to help the dying person to identify their needs and how the care provider can respond:

If you’re talking to somebody you can develop some rapport. Sometimes it happens really quickly; sometimes it happens over the course of many weeks. And you see them, you see who they are, and you can sort out with them what might be helpful for them.

Interviewees said that spiritual assessment approaches such as the Faith and Belief, Importance, Community, and Address in Care (FICA) Spiritual History Tool [38] or the SPIRIT history tool [39] may be useful to the provider if they identify, at the beginning of hospice palliative care, the background and characteristics of a person’s spiritual/religious life. When spiritual issues arise, these assessments may help care providers identify the resources and approaches that they may use as the person is dying. However, they generally provide only initial ideas about possible needs. A staff interviewee at the Salvation Army Agapé Hospice discussed how a dying person at the beginning of hospice palliative care may be very specific about his or her spiritual state and future wishes, but the person may well experience significant and unexpected thoughts, feelings, and needs that their religious or spiritual background is unable to resolve without further and perhaps different assistance than initially foreseen. A volunteer interviewee talked of instances of persons who had been raised and committed to a particular faith tradition doubting, in their last days and hours, their life-long religious beliefs and calling.

Putting Principle 2 into practice may be at odds with the expressed desires and patterns of practice of many health care providers who generally are trained to tap into their expertise and actively provide the range of options from which a person may choose. For example, in a recent survey of health care providers’ priorities for research in spiritual care in Europe [14], three of the top four priorities identified were ones that would seek to take control of, regularize, and professionalize the provision of spiritual care, using screening tools, models for conversation and spiritual care interventions.

What then is the best response that organizations need to promote and support a person-centered model premised on the dying person and the family leading the care? Organizations seeking to support high quality spiritual care need to be vigilant about and attentive to health care providers desiring routinized ‘interventions’ and ‘best practices’ approaches to spiritual care [40]. The practices of the hospice organizations in this study adopted, promoted, and enabled a more flexible, immediate, in-the-moment approach to listening and responding to spiritual needs.

These approaches included allowing time and patience for providers to develop rapport and trust within which deeper existential, spiritual and/or religious questions, and doubts, hopes, and fears can emerge. Some interviewees said, however, that, if the dying person is drawn to one of the care team members in particular, that person should be empowered to be especially watchful for these issues to arise. Further, if spiritual assessment tools are used, they should be seen as the beginning of a longer relationship and deeper dialogue – led by the person but nurtured by the care providers – about spiritual needs.

In addition to these specific preparations, Principles 4, 5 and 6 provide guidance to organizations about how to support care providers in creating an environment in which the spiritual issues are not forced, but emerge as the person journeys toward death.

### Principle 3

Palliative care is fundamentally a vocation, and the work is inherently spiritual.

Quality care of the dying involves skilled professional care and well-trained volunteers, and these care providers are expected to be accountable to defined norms of conduct and practice, either to their professional regulatory body or to the hospice palliative care organization they work for [23].

For spirituality to permeate the multiple domains of care, however, members of the care team will ideally go further than observing the norms of conduct and the rules of their profession or organization: in the hospices in this study, the care providers recognized their professional and organizational responsibilities, but they also conceived of their care work at least in part in spiritual or religious terms, and this perspective shapes their practice. In the interviews, people commonly described their work using the language of ‘vocation,’ a language that is explicitly or tacitly spiritual: ‘calling,’ ‘life’s work,’ ‘vocation,’ ‘service,’ ‘coming to hospice’—these were common descriptors among interviewees. Some interviewees talked about a revelatory or transformational experience, typically associated with loss, as their reason for doing hospice palliative work. Others talked about their work in terms of a quest for spiritual growth and development, or of a need to come to terms with the fact of death. Not all the interviewees’ experiences or expectations of vocation were all, strictly speaking, religious or spiritual. However, most people were of the view that these kinds of experiences were conducive to or welcoming of providing care in a way that is inherently spiritual.

Like Principle 1, Principle 3 is potentially controversial. If spirituality is the fabric binding the various aspects of hospice palliative care, the implication is that team members must be, at minimum, open to religion or spirituality in administering care and responding to the needs of patients, family members, and fellow care providers. And this openness must move beyond merely ‘calling in’ a specialist to deal with religious or spiritual concerns, but it does not necessarily mean that a care provider needs to have his or her own religious or spiritual beliefs, practices or commitments. One of the interviewees at the Zen Hospice Project declared that he is agnostic, but that he felt and reflected back to residents the attention to the spiritual that the Zen Hospice Project’s Buddhist foundation encouraged.

Discussions with interviewees that led to this Principle focused more on practical implications than for any of the other Principles. The fact (or requirement) that working in hospice palliative care is a vocational calling has implications in at least three areas: for recruitment, for the kinds of support given in the workplace, and for identifying potential problems that may emerge in the provision of care.

#### Recruitment

Since vocation and openness to spiritual concerns are important, there are potential implications for hospice palliative care organizations as they recruit staff and volunteers to provide care. The Zen Hospice Project volunteer program has a close association with the neighboring Zen Center, and managers of the program deliberately screen potential volunteers on the basis of a sense of vocation and calling. For recruitment of staff at the Zen Hospice Project and in the other hospices, there was not such an explicit screening process, but there was general agreement that the vocational element marks a good and desired staff member or volunteer, and those who are not ‘called’ generally leave the work after a short time. Whether or not there is an explicit selection process, the workplace environment tends to exert its influence to select those who see the work as vocational.

#### Workplace conditions and rewards

Understanding roles in palliative care in vocational terms has two implications for how the workplace operates. First, managers need to be aware that care providers may be looking for growth and opportunities to realize vocational (rather than or in addition to occupational, financial, or career) goals. One organizational response is to include a variety of spiritual practices as part of regular team meetings, and to offer training rooted in a spiritual tradition, as, for example, at the Zen Hospice Project, or to offer retreats that are oriented to religious introspection, as at the Catholic Hospice. The interview data suggest that many individuals across a range of roles avail themselves of retreat or ritual opportunities, speak highly of such experiences, and hold that such training and practice improves the quality of care they give. As an interviewee who previously volunteered and now was part of the staff of the Zen Hospice Project commented,

There is always room for improvement and the training showed me that there were things I could incorporate that would be helpful to me and benefit the patient.

At the Salvation Army Agapé Hospice, there are morning ‘huddles’ to bring people together to think about the dying residents’ needs, and there is, as noted above, a chapel service every weekday morning. A volunteer at the hospice said,

… I made sure I was there on [every] Friday morning before I did my volunteer stuff, to go in and take part in this twenty minute session and – non-denominational – and it was very good, very uplifting….

The second implication is that wages may not be a substitute for all the opportunities that care providers seek. Indeed, at the Zen Hospice Project, it was said that the nursing staff earn less than they would earn at an acute care hospital: their commitment to hospice is driven by more than money. Care providers in hospice palliative care may well seek opportunities for religious or spiritual practice. For example, the ability to take part in ritualized practices to provide solace and support for staff or volunteers who are grieving the death of a person may be an important part of the ‘package’ of rewards that are made available to staff and volunteers working in hospice palliative care.

#### Vocation and altruism

Interviewees raised the possibility of a conflict between their vocational goals and the interests of the people they are caring for. The fact that individuals are drawn to hospice palliative care may mean that the suffering of others becomes an occasion for one’s own spiritual goals and needs. As an interviewee at the Zen Hospice Project stated, summing up a common theme across the various hospices, ‘I gave them [the residents] something, they gave me something.’ Hospice palliative care is not entirely selfless work, and there is no firm line that objectively demarcates what is therapeutic for the dying person from what is beneficial for the care provider.

Some interviewees said that they are involved in hospice palliative care for altruistic reasons without regard to any benefit to themselves; for example, they have seen the beneficial effect of hospice palliative care in other people’s lives and deaths. However, some of the interviewees acknowledged and even celebrated the beneficial impact of their work on not only the dying person and on family members, but on themselves. Some spoke of their work as a way of coming to terms with their own experience of brokenness or fear of death. Others variously understood end of life care as integral to their spiritual path, as a sacred duty or mission, as an expression of deep commitment to social justice, as a way of coming to terms with their own mortality, and as a way of giving something back to others. One of the interviewees at the Jewish Hospice Program reflected on this theme:

This […] is a very giving job, time, etc., but every [dying] person has given me something of their life experience, they have taught me something and it has been an amazing journey. You are very sad when they pass away.

Another example of potential conflict of interests was mentioned by several interviewees: the serious prohibition against volunteer or paid care providers proselytizing. This prohibition is seen as one made on ethical grounds, not taking advantage of a person’s vulnerability as they near death, but some people may see the opportunity to provide guidance toward a particular religious view as the fulfillment of their own spiritual mission, and this potentially problematic aspect of spiritual care is commonly dealt with in general guidelines on hospice palliative care [41, 42]. This appears to be a difficult issue to balance, particularly when there are different family or care provider perspectives on the role of faith or spirituality. One family member interviewee, whose dying father had for significant reasons abandoned his faith, said,

In our lives, it’s very important to keep God first, God first in our lives. So I have also tried to drive that point home with my dad, and so going back to hospice, they have been very helpful with that angle.

For quality spiritual care, the data suggest that organizations need to identify, harness and celebrate the deep vocational commitment of those called to do the work. Further, organizations should be prepared to respond to a variety of needs and motivations of staff and volunteers [43], not uncritically assuming only selfless motivations. The true motivations may positively or negatively impact the quality of care. At the Zen Hospice Project, regular meetings allowed for care providers to identify issues themselves. There and in other hospices, there were opportunities for private one-to-one meetings with staff or other volunteers in which coaching and guidance were provided when the therapeutic effect could potentially be compromised by the benefit the provider was garnering.

The organizational responses to both the vocational dimension informing the lives of hospice care workers and the spiritual dimension of care calls for consciously and actively hiring and retaining care providers who are open to religious or spiritual care, or are religious or spiritual themselves. Quality care includes thinking about the spiritual needs and activities of care providers, and making sure that the providers’ needs are met. Quality care also demands setting up processes and safeguards within peer-to-peer or supervisor-to-staff relationships to guide and steer the sometimes turbulent waters.

### Principle 4

Quality spiritual care considers place as sacred.

Quality hospice palliative care involves tending to the activities of care providers at the bedside, ensuring that the proper person is performing the proper acts of care—tending to questions of ‘who’ and ‘how.’ The interviewees in this study also point to the centrality of ‘where,’ to the kinds of spaces and overall atmosphere of the hospice.

In the literature, place is sometimes explored in relation to how care and healing [44, 45] can be impacted by factors such as architecture, the use of lighting and sound, the arrangement and kinds of furniture and objects in a room, the provision of spaces or rooms for art and music, the use of natural environments such as gardens [46, 47]. In the hospices visited, there was a concerted effort to create a special sense of place, based on a conviction that the place where one dies ought to be, in religious language, ‘sacred’ or ‘holy.’

At the Zen Hospice Project, a sense of place is created in part through the deliberate use of language, with the hospice being referred to as a ‘guesthouse’ and patients as ‘guests.’ (The building is, in fact, a Victorian era home in a residential neighborhood, next to schools and cafes, homes and businesses.) ‘We can’t quite touch it,’ said a staff interviewee at the Zen Hospice Project, ‘but we can talk about it. We know it’s there. People step into this building, they say they can feel something.’ Another staff interviewee said, ‘[This place] is conducive to compassionate care, beautiful care. It is a “house-church.”’

Gardens also contribute to the sense of place, and provide opportunities for coming together. At the Salvation Army Agapé Hospice, when residents and their families can go into the garden, a staff member interviewee said that there is,

… a sense of peace and a sense of community… and even if the resident isn’t interacting, is imminently dying, there’s still that togetherness around that person, and the garden creates that, the garden provides that intimacy, and people connect with nature.

Objects can also enhance a space and open it up for discussion about spiritual issues. A volunteer interviewee at the Catholic Hospice said:

I’ve brought religious objects, medals – you’ll see statues and things around the house so you know the person finds them significant – so I’ll bring a religious object, a cross, or a picture with a prayer on the back. They seem to value that a lot.

Words such as “religion,” “spirituality” and “sacred” have multiple meanings. As noted above, in this study a broad view approach was taken in conceptualizing “spirituality.” Principle 4 invokes the term “sacred,” but one might reasonably ask what this word means. A useful approach is found in the work of Ann Taves, who, in describing religion, skirts the question of a definitive first order term, opting rather for a generic quality that Taves describes as ‘making special.’ Religious-like phenomena, Taves argues, are characterized by the effort to mark them as special or extraordinary, and there a variety of ways specialness is generated: the use of ritual and ceremony, or setting aside some thing or quality outside the sphere of commodification [48]. Similarly, Ellen Dissanayake [49] has argued that the core of art is a process of making things special, premised on a fundamental cognitive and emotional distinction between an ordinary, mundane realm and one that is inherently unusual (or supernatural or transcendent or otherworldly). In searching for a core feature to describe the not strictly utilitarian nature of art, Dissanayake [50] settles on ‘specialness’:

The best word for this characteristic of the arts seemed to be special…. While “special” might seem too imprecise and naively simple, or suggest mere decoration, it … denotes a positive factor of care and concern that is absent from the other words. It thus suggests that the special object or activity appeals to emotional as well as perceptual and cognitive factors—that is, to all aspects of our mental functioning.

As illustrated above, interviewees in this study indicated that treating the place of death as special in this sense creates an environment in which spiritual issues more readily emerge, and care providers can more readily respond in terms of spiritual care. Generally, the place of dying needs to be imbued with qualities that transform space into place. In the tradition of phenomenology and humanistic geography, space is what we move through, whereas place is where we live, where we dwell [51]. Just as narrative takes a basic chronology of events and turns it into a meaningful pattern or plot, architecture, objects, stories, and gestures transform flat, mathematical space into lived, meaningful place. Place is created through a variety of means: architectural design, the arrangement of a room, symbolic objects, rhetoric and stories, gestures, ceremonies, memorials, rituals – each of these is constituent of place that is ‘conducive to compassionate care, beautiful care.’

Principle 4 requires that the planning, organizing, and delivering of spiritual care be closely attentive to the details, qualities, and aesthetics of the place in which the person is dying. As we know that most people, given the choice, prefer to die at home, hospices or palliative care wards should, to the degree possible, be made to feel like home. Particular locales are associated with unique moods and feelings. Research has demonstrated that environments can be experienced as ‘either authentic or inauthentic,’ as ‘caring and connecting’ or as promoting ‘spatial separateness and isolation’ [52]. Though improvements are being made in health care settings through tending to matters of architectural design, furnishings, and use of space, a concerted effort in tending to place is part of the culture at the hospices in this study.

### Principle 5

Quality spiritual care requires care providers to allow spiritual questions and issues to emerge in the course of developing relationships with the dying and their family members.

In a response to organizations’ need to support their providers to be directed and guided by dying person and the family in matters of spirituality and spiritual care (Principle 2), the fifth Principle may provide some ‘unstructured structure.’

Because the importance of spiritual issues ebbs and flows over the period of dying, the evidence from the hospices in this study establishes that it may not be possible to predict—and it may not be appropriate to provoke – spiritual questions. Instead, one interviewee, a staff member, described the emphasis of his approach as ‘being with’ and ‘walking with’ the person who is dying or a family member, and then “facilitating for them” the understanding of the issues they are facing. He said, rather than asking questions directly, “reading the larger situation” can provide guidance to the care provider on how to be of assistance:

I believe that once you get to know someone, there are many ways you can get an inkling in terms of how they are feeling and how they are doing, how things are going, from reading the larger situation.

Interviewees told us that chaplains in hospice palliative work sometimes have to work at not emphasizing their role in spiritual care. A staff member at the Salvation Army Agapé Hospice, for example, commented,

I try to see each resident who comes in within the first day or two of their arrival here, and people will often say, “Oh, I’m not spiritual.” “Well, that’s all right. I’m here to see how you are. Tell me how you’re doing; I just want to know how you are.” From there a relationship forms or doesn’t form, depending on what their will is.

Another staff interviewee at the same hospice spoke of the role of hospice palliative care providers as ‘a ‘ministry of presence’; [this] means being present, listening.’

A volunteer at the Catholic Hospice said that in this attentive listening and waiting, his and other care providers’ own spiritual interest should not be at the forefront:

Your presence as a human being with a spiritual inclination is very helpful to the person without verbalizing it too much. And I think you can convey your concern for their pain without even thinking about it too much. In fact, the less you think about it, probably the better.

A care provider at the Zen Hospice Project noted that it ‘is also important that residents feel a volunteer’s spirituality, not necessarily know about it.’

Care providers are often trained to be proactive and task oriented, and so simply being present and listening is often perceived as not being active enough. While some people may have a specific request that can be acted on, it is often the case, as a volunteer at the Salvation Army Agapé Hospice noted, that patients

don’t want answers. They don’t want us to figure things out for them. They just want to talk…and boy do they want to talk. I think good listening here is what is required.

Listening, being present, and engaging in conversation are the means to build rapport, and out of rapport spiritual needs and potential responses emerge. While interviewees consistently said that attentiveness to expressed (though possibly oblique) questions and needs is essential to organize and enable delivery of spiritual care, interviewees also highlighted that there are tensions created by a felt need to be active. In particular, health care organizations and providers are under constant pressure to perform measurable routines and tasks.

One of the interviewees ironically caricatured his practice and listening as ‘doing nothing, really well’—the irony is injected because listening and waiting can be at odds with the pressure within the health care world to *do something*. Accordingly, it is important for organizations to recognize, and specifically make provision for, the time it takes for the spiritual to emerge. Listening, being present, quietly observing—these are efficacious acts in and of themselves, and should be valued as such. Some interviewees said that training, mentoring, and practice contribute to the development of the skill of being present, waiting and listening, and being present needs to be valued as a deliberate form of care. They said that a quick diagnosis of spiritual needs is unlikely to be enough, as insight into spiritual needs requires time. In organizing quality spiritual care, therefore, institutions need to ensure providers are comfortable with silence and listening, and have the time to do so.

There are some challenging cultural assumptions here regarding what it means to be in control of or responding to a situation. In Taoist terms, the ethos is that of *wu wei*, the subtle, paradoxical art of doing nothing, but in a deliberate, though effortless, spontaneous, and natural fashion [53–55]. The language of ‘interventions’ through a directed agency tends to dominate medical discourse; the idea of *wu wei* suggests an alternative. *Wu* (no) *wei* (action) is sometimes translated as ‘non-action,’ but it is better conceived as a particular kind or style of action variously described as ‘following along with the way things naturally are and not adding any human effort’ [53]; ‘not interfering in any way at all with the course of things in the world’ [54]; or as ‘refraining from activity contrary to Nature.’ [55] The hospices in this study tend to model a course of action that is closer to a conception of ‘non-action’ than to deliberate, focused, interventionism.

This Principle also means providing encouragement and reward to care providers for the development of relationships with those who are dying so that there is the necessary comfort level for the spiritual questions to emerge at some point in the hospice palliative care trajectory. It may also involve developing and ensuring the opportunity for volunteers to spend the necessary extended time with the people who are dying, but also incorporating significant effort to ensure that there is good communication with the paid care providers so that as they perform their task-driven work, they can attend to the spiritual needs in a responsive way. While it is perhaps going too far to frame the interaction between care providers and patients in terms of friendship, the interviewees repeated mention of ‘relationships’ with dying and family members points to the inadequacy of an ethic only founded on duty and responsibility.

### Principle 6

Quality spiritual care entails the act of ‘witnessing.’

It is generally understood that quality end of life care involves active assessment and treatment, but also contemplative, reflective care [22]. The interviewees suggested that in addition to these aspects, quality spiritual care at end of life is rooted in the act of ‘witnessing.’ As one interviewee at the Zen Hospice Project put it, reflecting a link with Principle 2:

our radical openness to meeting the person where they are [involves] present[ing] a clear witness which is supportive and liberating for the resident.

The word ‘witnessing’ may be seen to have a Christian orientation, but in the interviews, ‘witnessing’ and similar language— ‘waiting,’ ‘walking with,’ ‘accompanying’—were used by interviewees of all faiths to describe the atmosphere created in an extended period of attentive care. They used language about seeing the person who is dying and the family through a liminal time in their lives. The word ‘liminal’ comes from the Latin *limen*, which means ‘threshold,’ and thresholds mark transitional zones between different kinds of spaces. Metaphorically applied to the lifecycle, liminal periods are those transitional times in the course of a life [56]. Death is a time of liminality, and witnessing is that quality of care that is attuned to a situation of transition and transformation.

The interviewees said that witnessing entails several dimensions. At its barest, it involves ensuring, if at all possible, that individuals not pass through the liminal time alone. One interviewee (a member of the clergy) said,

I believe all people have that insecurity, you know, “have I fulfilled my potential?”, you know, “what has God wanted for me in this world?”, you know, “will we be able to measure up to the expectations of our potential?”

In such reflective moments, a looking back and a looking forward, the interviewee said that it is necessary to

…flesh out things that are meaningful and significant, in a way that can help…. It’s about taking each person, walking with them, facilitating with them … a greater understanding of what’s going on.

Witnessing is also important to share the perspective and wisdom of having seen and experienced death with those who remain after a person who has died. One interviewee (a cleric) said,

I went there, and I held the patient in my arms. It was a three year old girl with cancer. Then 30 minute later, the nurse [said], “Deacon, 315 just expired.” It was a Haitian lady, 103 years old. Three years, 103 years. What can you reflect about that? Talking to the family of a three-year-old, talking to the family of a 103-year-old? God provides: I don’t know what did I say, but they got the message.

Finally, there is the importance of witnessing on behalf of the larger community, to honour the life of the person, and to mark and acknowledge the death by being present. An interviewee, a volunteer, at the Zen Hospice Project reflected:

I think, what if there is no heaven? What if there is no afterlife? And this is just me in the room, you know, honouring this person’s life lived, and what remains of them is this physical shell, which is very still and very cold, and it’s still worth honouring, even if that’s totally it. It’s a really sacred time, and to bear witness to it is worth doing. Even for me, even if I’m the only person witnessing this, *I’m* witnessing it.

There was a similar example of marking the life and death formally with a larger group specially assembled to do so at the Salvation Army Agapé Hospice. There, the person’s life and the transition to death and beyond is marked by a group of people verbalizing their memories in a group, to acknowledge that while the person is not physically present (the body has been removed from the space), the memories and impact of the person remain.

The emergence of this theme of witnessing was something of a surprise in reviewing the interview data. Spiritual and religious care in the context of hospice palliative care refers to a host of practices and attitudes, but typically the notion of witnessing is not among them. In the literature, witnessing has been framed as ‘as existential position in caring;’ it involves being present for a person in an ‘existentially demanding situation’ (end of life or extreme suffering) and entails ‘perceiving a person’s deepest needs and desires.’ [57] Others describe ‘witnessing’ as a ‘consoling presence.’ [58–61] The interviews extended the concept of witnessing well beyond these notions.

What are the organizational practices that support witnessing? In these hospices, staff and volunteers were permitted and encouraged to spend the necessary time to witness the emerging questions or struggle or acceptance of death, to attend at the time of death, and to reflect not only on the death but also the lives of the persons who have died. The care providers not only offered care—they watched, experienced, remembered, reflected on, and recounted aspects of the person’s life, the person’s journey to death, the death itself, and, where appropriate to the person or the family, the person’s expected experience after death. Not only was time allowed, but the interviews showed that the care providers had been encouraged to reflect on and express and describe (in appropriate settings) the transition they were witnessing. Finally, as was most evident in the last gathering at the Salvation Army Agapé Hospice, the organization established and encouraged rituals that help to structure, formalize and sustain the practice of witnessing.

### Principle 7

High quality end of life spiritual care includes rituals to ensure space, time, and opportunities to mark transitions and process experiences.

In many of the interviews, there was a considerable emphasis on a variety of actions and activities that are repeated on a regular basis by many of the health care providers and volunteers. Further, it emerged that these repeated actions provide a rhythm to the work of caregivers and others. These rhythms function at three different levels.

First, within each organization, there are consistent and meaningful activities for groups of staff and volunteers to structure the conduct of day to day work. Second, there are distinct patterns identified in the practices of individual care providers to mark transitions between different spaces and moments in their workday. Third, there are repeated actions that are used to mark the arrival and departure of patients, to honour their lives and mark their deaths.

With respect to repeated actions at the organization level, there are activities repeated on daily, weekly, and yearly cycles. At the Salvation Army Agapé Hospice, the daily non-denominational prayer service in the morning provides a time for any staff member, volunteer, family member or person who is dying to join with others for prayer, silence and song. A daily ‘huddle’ practiced at the same hospice is part business meeting, part reflective practice, offering a formal, daily gathering for those affecting and affected by hospice work, another structured moment to pause and reflect on the day’s course of events. Similarly, the Zen Hospice Project marks changes of volunteer shifts with a short ‘sitting’ period, a form of quiet meditation preceding a period to share feelings, thoughts, and concerns. The director of the volunteer program described it this way:

In a shift change meeting, we will start off with a 10–15 minute sitting period, so everyone, the volunteers leaving their shift, the volunteers coming in for theirs, are sitting together. And then we begin basically a process circle where each individual volunteer has an opportunity to check in on their experience.

Such a sitting practice serves as a transition for volunteers, helping to ensure an opportunity to gather oneself and share needed information. Though focused on volunteers, other staff commonly attend. At the Catholic Hospice, regular business meetings start with a prayer, a short period of silence, and a remembrance of those who have died recently in the hospice’s care. These various formalized meeting periods share the common aim of shift-change meetings, namely, the dissemination of practical information, but their formal, ritualized qualities also serve to encourage and offer reflective and therapeutic practice for care providers, as well as mark the work as special.

Interviewees in each the hospices also noted a series of activities that they undertake individually or collectively to mark different stages in the course of caring for patients. Most care providers employed more or less personal or private acts in the context of transitions between the rooms of different patients, or in marking transitions in the workday. The very layout of the Zen Hospice, with guests upstairs, kitchen and living room (chiefly space for caregivers, volunteers, staff and family members) on the main floor, and offices (for administrative work) downstairs, serves to demarcate shifts in attitude, demeanor, and intentional focus. The stairs leading from shared domestic spaces to the rooms of the guests function to create a transitional space and time for reflexive movement and shifts in purposes and objectives, as care providers travel up and down the stairs. A similar layout is employed at Salvation Army Agapé Hospice. A volunteer described her ritualized use of breath in moving from room to room:

And I suppose my preparation, some of it – I don’t always succeed at this – but what I try to do – and this is not my idea, it’s something I read somewhere: before I go from one room to the next room, as I’m cleaning my hands, usually, I try to leave whatever was in that back room and go into this new room … having taken three deep breaths and just walking in and hopefully not dragging either the issues from the other room or maybe my personal issues that day, into that room. Just try to go in like a breath of fresh air…

A nurse at the Zen Hospice Project related a similar practice. In meditative traditions, a focused, stylized use and control of the breath, coupled with various gestures, bodily comportment, special objects, and set-aside places and times serve to ritualize a most common, instinctual act. Ritualizing the breath aids in the slowing of the heart rate, calming agitation, and focusing attention on one’s surroundings and matters at hand.

Thirdly, the hospices visited have developed passage rites of their own, marking the entrance and exit of patients. That such practices have developed indicates a desire or need to mark as special these moments in the course of an individual’s life, while simultaneously inscribing the work and the place of work with more than ordinary meaning and significance. A staff member at the Salvation Army Agapé Hospice described a passage rite:

If we’ve had a resident who has been here for a long time, the room that that person has been in when they pass, we gather the staff together, and we have a period of time, a little ceremony that we do, where we do ‘beginnings and endings’, and it involves some scripture, it involves the lighting of a candle, it involves giving the staff time to share their thoughts and memories about this person, and it ends with a prayer for the individual and for a cleansing of the room so that it’s prepared for the next.

An interviewee at the Zen Hospice Project, a nurse, described their practice of washing the body of the deceased:

… one [ritual] is the bathing of the body in these ancient oils. So you bring the water up with all these washcloths and then the family, friends, whoever wants to participate, volunteers, staff, we all gather round the bedside and usually there’s one spokesperson – it could be a volunteer, or could be [the director of volunteers], who will be the person who kind of guides people and encourages stories … as you … dip the washcloths in these ancient herbs, and tenderly wash the hands, or the face, or the feet.

At the Salvation Army Agapé Hospice there is a bereavement coffee hour for families and care providers every other month and a formal memorial service twice yearly. The Catholic Hospice organizes and performs an annual memorial mass, inviting family members of those who died in the previous year.

A short definition of ritual is *prescribed, embodied action*: prescribed because ritual exhibits formality, repetitiveness, and continuity; embodied because it is overtly a practice of the body, taking place through overt gestures and acts [56]. Faith traditions often have particular practices, ceremonies, or rites that will ideally take place leading up to, at, and after the time of death. One of the commonly offered solutions to this question of how to provide for people of different faith traditions is short descriptive accounts of the beliefs and practices of these traditions, and there is considerable literature about the variety of religious and ritual requirements and needs present in today’s ethnically and religiously diverse society [62–64]. Certainly the insights from this work are necessary and valid; the hospices participating in this study indeed aim for multicultural awareness and sensitivity. Nonetheless, the nature of the rituals discussed here clearly goes beyond these formal religious practices.

The anthropologist Victor Turner argues that in the context of life transitions ritual is potentially transformative insofar as it allows the ‘the contents of group experiences [to be] replicated, dismembered, remembered, refashioned, and mutely or vocally made meaningful.’ [65] Ritual theory and everyday language refers to these transitional moments and practices as ‘rites of passage:’ birth, coming of age, marriage and death. Essential to rituals and rites of passage theory is the notion of transformation. Society is composed of a set of recognized status positions, and the work of passage rites is to move individuals through these (often age related) social positions by transforming them from one state to another, for example, from adolescence to adulthood, from being single to being married [56].

Passage rites aim to move individuals from one status or state to another, but they are not only aimed at the individual: passage rites also have a public and social dimension [56]. Therefore, the rituals at all three levels in these hospices (the organization, the care providers, the dying and their family) were effective at something more than just structuring the work of the hospices. They are best seen as an inherently essential element of care and an environment in which spiritual care attends to and provides an active presence during the transitional and transformative time associated with death.

In quality hospice palliative care as with other areas of health care, operational procedures and routines provide structure and consistency in the delivery of care. In quality spiritual care, the interviews in this study indicate rituals and routines provide a foundation and a constant reminder and call to spiritual issues. The functions, effects and outcomes of ritualizing end of life care in these ways ensures there is a sense of community that enables spiritual care, created through traditions. It also ensures that the people who are dying – their lives and their deaths – are honoured, and that the hospice and the people caring in it, can, and do, maintain personal and collective and institutional relationships to the dead. Finally, the rituals develop a form of institutional memory that preserves the persons, outside the push and pull of a more medicalized, secular sphere.

### Principle 8

Quality spiritual care involves creating and sustaining relationships beyond those typical between co-workers.

Quality end of life care involves high levels of coordination of activities of various providers. The Canadian Hospice Palliative Care Association’s *A Model to Guide Hospice Palliative Care* says that, the interprofessional team members ‘collaborate and share information to promote continuity and enhance care delivery to the person.’ [21]

Effective, respectful collaboration is a necessary condition for quality care; but the interviews reveal that such collaboration is insufficient, that there is something more than professional collaboration in high quality spiritual care. The interviews reveal that the experience of working with the dying and their families tends to create bonds of affection and sympathy with co-workers beyond that generally formed in other work environments. Interviewees consistently reached for the language of kinship (‘family,’ ‘marriage’), group solidarity (‘community’) and affective bonds (‘friends,’ ‘love’) in describing relationships to co-workers.

A volunteer at the Salvation Army Agapé Hospice said,

Wednesday is probably my best day of the week. I like coming here, and it’s not just what I do, it’s because of the people around me when I’m here doing what I’m doing.

A volunteer at the Jewish Hospice Program in Toronto talked about her experience when attending training events:

And in speaking to other people, so when you come here for the training, you’re not only being trained about whatever it is, you are speaking to colleagues going through the same thing…. It validates what you’re going through because your spouse doesn’t get it, your friends don’t get it, your family don’t get it. It’s a very unique position we’re in.

The rabbi in the L’Chaim Jewish Hospice Program at the Catholic Hospice said,

Having everyone say when you are walking into the building, “Hi, Rabbi, how are you? Hi, Rabbi, how are you?”, you know, with a smile, and it’s not forced: it makes me feel good, the fact that I have the same feeling for them, and it’s not forced. I love everybody in this building. They’re great people to work for, they’re great people to work with.

The hospices in this study demonstrated a clear commitment to foster deep relationships among co-workers, beyond the trust and professional collaborative and cooperation called for in the Canadian Hospice Palliative Care Association’s *Model Guide* [23]. They enabled these relationships through leaders consciously modelling, and explicitly establishing expectations for, mutual care among co-workers. The rituals that they have established that gave rise to Principle 7 also provided a platform for a sharing of experiences that brings co-workers closer together on a personal as well as professional level. The hospice organizations also provided regularly opportunities for retreats and commemorative events, providing further occasions for volunteers and staff to share their perceptions of the depth of meaning and impact of their shared vocation; indeed, such opportunities instil the institution with an ethos of vocation.

### Principle 9

Quality spiritual care emphasizes the role of volunteers; the presence and work of volunteers reinforces and ensures that hospice palliative care is grounded as both vocational and spiritual.

Models of best practice in hospice palliative care generally recognize the importance of the contributions made by volunteers [23, 66, 67]. Not only did the interviews in this study support research arguing for the effectiveness of volunteer programs in end of life care, the interview data suggest that a volunteer program is an essential element in creating quality spiritual care.

Interviewees at each of the hospices emphasized that a portion of daily operational tasks and caregiving falls to volunteers; beyond this practical dimension, however, is the observation of the essential role being played by volunteers in establishing the success and quality of overall hospice palliative care. Following are four quotations from the interviews that illustrate the pivotal role played by volunteers.

…the volunteer presence […] is really wonderful for the residents…

I cannot say enough about our volunteers…. They have time to deliver the care that is needed (outside medical care) and residents open up to them in ways that they will not with staff.

Really, honing in on what the volunteers do. Volunteers are amazing people, who give their entire heart to this organization; it’s quite inspiring to watch their contribution to the community and … the little things that they provide.

Volunteers provide a passion that you don’t [always] see in employees, they are here even if they don’t get paid…. And they have heart, ….

The presence of volunteers provides a diffuse sense of the commitment demanded by hospice palliative work; their presence gives encouragement to paid staff and residents alike. But beyond general clinical care, clinicians, nurses, and other professionals are often thought not to have the time, inclination, or training to deal with the spiritual dimensions of end of life, and, as a staff interviewee said, “…often the burden and blessing of spiritual care is assigned to volunteers.” Thus, when thinking through the role of volunteers, they can often be seen as essential to the provision of spiritual care by the entire team of care providers. A volunteer interviewee at the Zen Hospice Project commented that volunteer presence effects awareness of

being versus … doing. You know, if there’s a nurse in the room, they’re doing something because they are so short-staffed; they have a long list of things to do. And if they don’t have anything to do in the room, they’re probably not going to be there. So a volunteer can be the person who can just – I have heard it said, “you Zen Hospice volunteers are so good at doing nothing,” which is kind of why we’re there. It gives this extra cushion, buffer, to the suffering situation, because there’s somebody there who … could be there at their bedside for as long as they need you.

Since volunteers are providing their time for free, the care they offer is felt to be from the heart, a felt experience that contributes to a general environment of attentive commitment to spiritual care and concerns. ‘I’ve often felt,’ said the director of volunteers at the Zen Hospice Project,

that the presence of the volunteers is a reminder to the nurses that, “Oh, I can tap into my spiritual nature in addition to just focusing on the clinical circumstances of the resident whom I am serving or treating”…. And so if you take the volunteers completely out of the picture, I could see how, given the intensive nature of this work, for a lot of nurses, they might start to lose touch with that more spiritual approach.

Operationally, therefore, quality end of life spiritual entails placing volunteers at the forefront of institutional organization and delivery of care. Resources need to be committed to recruiting and training volunteers, and to periodic retreats and workshops that build on the initial training. The Zen Hospice Project in particular has developed a comprehensive volunteer recruitment and training program. The administration and organization of care should actively involve and place volunteer work at the centre, not the periphery, of care. Opportunities for volunteers to interact with administrators, clinicians, nursing staff, patients, and family members should be encouraged. At the Zen Hospice Project, the executive director, who is also a physician, noted that clinical care needs to be effective and efficient precisely in order to make space and time for the provision of spiritual care offered by all care providers, including volunteers. Recalling the first Principle, the work of volunteers needs to be consciously integrated into the health care teams in an explicit fashion. One staff interviewee at the Salvation Army Agapé Hospice commented, ‘Don’t silo the volunteers. They are part of the care team.’

## Conclusions

This study began with the observation that incorporating spiritual care in end of life care in secular settings is difficult, due to the medicalization of dying and death and the nature of support for health care from the modern secular state. In the interviews at the four hospice organizations with religious and spiritual foundations, the nine Principles emerged; reflecting on and reviewing the interviews from the perspective of these principles, there is no indication that interviewees felt that their own organization was necessarily performing well on each and every one of the nine. This suggests that even when there is a specific commitment to providing spiritual care at the end of life, the task is daunting. Nonetheless, the nine Principles serve to generalize themes found across the interview settings, and they may provide guidance for secular health care organizations who wish to improve the quality of the spiritual care they provide.

The first Principle, incorporating the spiritual into every other aspect of end of life care, comes up against the health care system’s divisions of labour and relatively distinct domains of disciplinary expertise in the provision of care. Nonetheless, this research has shown that with support from an organization, it is possible and desirable that the spiritual become a defining feature of the kind, nature and quality of all end of life care. This is especially so given Principle 2, which states that unlike other areas of care for which there are “interventions” and opportunities for shared decisions about options provided by medical science, spiritual care needs to be guided and directed by the dying person and his or her family. We argue that this is achieved when organizations promote and enable care providers to have a flexible, adaptive, immediate, in-the-moment approach to responding to spiritual needs.

Principle 3 says that hospice palliative care is fundamentally vocational (and hence inherently spiritual), and so provides a positive foundation for engagement and passion upon which hospice palliative care organizations can build as they work to improve the quality of spiritual care. However, our findings point to a need for organizations to attend to some practical issues that arise with respect to recruitment, workplace conditions and rewards, and the thorny questions that may arise if care providers’ care for the dying may conflict with their own personal needs.

Principle 4, requiring that spiritual questions and issues be allowed to emerge, recognizes that the spiritual issues ebb and flow as a person is dying; as a result, early assessments may become less relevant as the person encounters the spiritual. This principle requires care providers to wait, listen and be carefully attentive, rather than to actively provoke or enact interventions. We argue that organizations should avoid requiring and overemphasizing formal assessments and ‘doing’ spirituality. The fifth Principle, incorporating ‘witnessing,’ is consistent with the practice of allowing spiritual questions to emerge, and requires that the hospice palliative care organization enables care providers to be present at the time of death, to bring to a dying person and his or her family their wisdom of having witnessed death before, and to honour the life of the deceased person and mark the death on behalf of the wider community. Principle 6 emphasizes that “place” is sacred, and points to the necessity of creating environments in which spiritual questions and answers are more likely to both emerge and receive resolution. This study suggests strongly that organizations wanting to raise the quality of the spiritual care should enable care providers to adjust and adapt space to make it a special, sacred place, including arranging room layout and design, and the use of symbolic objects, music, artwork, and elements from nature.

Principle 7 insists that rituals and time for processing experiences are necessary for high quality spiritual care. The rituals – aside from religious rituals that may also be desired – should be at the institutional level (for example, when opening meetings), at the point of care (the provider’s personal ritual practices), and for patients and their families to honour lives and mark deaths.

To support and sustain the ethos of spiritual care, Principles 8 and 9 would require a hospice palliative care organization to encourage close, even family-like, bonds among care providers and other staff, and to incorporate and celebrate the altruistic offerings and presence of volunteers as a constant reminder and confirmation to paid staff of the vocational and spiritual nature of their own work.

Individually, each of these Principles has been attended to in the general literature on hospice palliative care and sometimes the literature on spiritual care at end of life. However, the first major contribution of this study is to tease apart some of the underlying mechanisms and subdivisions of activities and concepts. For example, while the role of volunteers is generally thought to be to supplement – at low cost – the efforts of paid staff in hospice palliative care organizations, this study has identified that the *presence* and not only the *functions* of volunteers can contribute to an ethos that is conducive to high quality spiritual care.

This study’s second contribution is to bring all nine Principles together as a potential multi-pronged framework for improving spiritual care, along with some organizational practices that could support each of the Principles. This set of nine Principles invites uptake by organizations to see how moving toward them brings more comprehensive, higher-quality spiritual care. Future research possibilities include tracking organizations’ experience bringing these Principles to life, and identifying, documenting and evaluating further examples of organizational practices that are in line with the Principles.

## Additional file

Additional file 1: Question Sets for Interviewees. The interviews were conducted with the questions in this file in hand, as primers for a conversation. These were potential questions, and the entire set was not asked in each case, and not in the order listed. (PDF 85 kb)

## Footnotes

1 For this study, ‘care provider’ is defined as the Canadian Hospice Palliative Care Association [23] defines formal caregivers: ‘Formal caregivers on the team may include, but are not limited to: chaplains, dieticians, nurses, pharmacists, physicians, psychologists, social workers, speech pathologists, integrative therapists, occupational therapists, physiotherapists, recreational therapists, volunteers.’
