# Supplementary material for: Organization-level principles and practices to support spiritual care at the end of life: a qualitative study
Source: BMC Palliat Care. 2017 Apr 11;16:24. doi: 10.1186/s12904-017-0197-9 (PMC5387203; doi:10.1186/s12904-017-0197-9)
Supplement: Additional file 1: — Question Sets for Interviewees. The interviews were conducted with the questions in this file in hand, as primers for a conversation. These were potential questions, and the entire set was not asked in each case, and not in the order listed. (PDF 85 kb) [file 12904_2017_197_MOESM1_ESM.pdf]

Additional File 1 – Question Sets for Interviewees

The interviews were conducted with the following questions in hand, as primers for a conversation. These were potential questions, and the entire set was not asked in each case, and not in the order listed here. The aim was to engage the interviewees in conversation about the their experiences and thoughts, eliciting stories and vignettes about their experiences with the care they received or provide, and some of the specific examples of care that have been satisfactory and some that have not been satisfactory, from their perspectives.

For patients and family members

1. How have clergy (pastors, priests, imams, etc.) responded to your illness?
2. How has your family responded to your illness?
3. Are you worried or concerned for your family?
4. Are you able and how often do you attend religious services?
5. Is there a part of your religious/spiritual life made more difficult as a result of your illness (liturgy, worship, ritual, for example)?
6. Does religion/spirituality play a role in your understanding your illness?
7. Does religion/spirituality play a role in your family's response to your illness?
8. Do you read scripture (sacred texts)?
9. What words do you use to denote or describe the religious (e.g., sacred, holy, feeling the spirit)?
10. Have you had any specific religious/spiritual experiences in your life?
11. What places (things, objects, times) are sacred to you?
12. How public/private is your spirituality?
13. How did you learn of your illness?
14. What are the big questions in your life right now?
15. What are your views of death?
16. Do you find comfort or strength in your religious or spiritual life?
17. Do you think at all about funerals?
18. What is important to you when you imagine your own funeral.
19. Do you feel (hopeful, loved, connected, a sense of purpose).
20. What are you thankful for in the course of your day?
21. Do you feel you've led a productive, meaningful life?
22. Are there any particular prayers, stories, or rites that have taken on new meaning for you?

For Administrators and Care Providers

1. How does spirituality shape the understanding and provision of palliative care at the hospice?
2. What is unique about a (Muslim, Buddhist, Catholic, Jewish, etc.) hospice?
3. Is the hospice grounded in any particular religious principles, beliefs, attitudes, or practices?
4. Are there needs and expectations particular to patients from a specific religious tradition?
5. What constitutes a good death in (Islam, Judaism, Buddhism, etc.)?
6. What are the obstacles in providing spiritual or religious care to palliative patients?
7. How does your religious or spiritual life impact your hospice work?
8. Does religion/spiritualty aid in grief and bereavement?
9. From your perspective, how might palliative care in secular-oriented health systems learn from hospices?
10. How is the hospice funded?
